# Exploiting and assessing multi-source data for supervised biomedical named entity recognition

**DOI:** 10.1093/bioinformatics/bty152

**Published:** 2018-03-10

**Authors:** Dieter Galea, Ivan Laponogov, Kirill Veselkov

**Affiliations:** Computational and Systems Medicine, Department of Surgery and Cancer, Faculty of Medicine, Imperial College London, London, UK

## Abstract

**Motivation:**

Recognition of biomedical entities from scientific text is a critical component of natural language processing and automated information extraction platforms. Modern named entity recognition approaches rely heavily on supervised machine learning techniques, which are critically dependent on annotated training corpora. These approaches have been shown to perform well when trained and tested on the same source. However, in such scenario, the performance and evaluation of these models may be optimistic, as such models may not necessarily generalize to independent corpora, resulting in potential non-optimal entity recognition for large-scale tagging of widely diverse articles in databases such as PubMed.

**Results:**

Here we aggregated published corpora for the recognition of biomolecular entities (such as genes, RNA, proteins, variants, drugs and metabolites), identified entity class overlap and performed leave-corpus-out cross validation strategy to test the efficiency of existing models. We demonstrate that accuracies of models trained on individual corpora decrease substantially for recognition of the same biomolecular entity classes in independent corpora. This behavior is possibly due to limited generalizability of entity-class-related features captured by individual corpora (model ‘overtraining’) which we investigated further at the orthographic level, as well as potential annotation standard differences. We show that the combined use of multi-source training corpora results in overall more generalizable models for named entity recognition, while achieving comparable individual performance. By performing learning-curve-based power analysis we further identified that performance is often not limited by the quantity of the annotated data.

**Availability and implementation:**

Compiled primary and secondary sources of the aggregated corpora are available on: https://github.com/dterg/biomedical_corpora/wiki and https://bitbucket.org/iAnalytica/bioner.

**Supplementary information:**

Supplementary data are available at *Bioinformatics* online.

## 1 Introduction

Publications in the biomedical field have increased considerably over the years, with over 27 million total publications contained on the PubMed repository alone. With increasing information resources, searching and extracting valuable information has become more challenging using traditional methods. This has led to an increased interest and need of text mining systems that automate information extraction. Named entity recognition (NER) is a critical step in such workflow, classifying sequences of words to specific classes. In biomedical named entity recognition, this involves identification of biological/chemical entities such as genes, proteins, chemicals, cells and organs from unstructured text.

Several approaches have been developed and employed throughout the years to perform this task. Dictionary lookup is the simplest approach and is used by literature mining tools such as PolySearch (Liu *et al.*, 2015). While the advantage of this is that annotated data is not required for training (although required for evaluation), this approach often suffers from low accuracy due to its inability to disambiguate words based on context or semantics. This requires further pre- and post-processing steps, which are often hand-crafted rules. Furthermore, such approaches are limited by the availability of a ‘complete’ dictionary, therefore are unable to adapt and identify new or unseen entities.

The GENIA project (Kim *et al.*, 2003) was amongst the first major efforts for the development and optimization of machine learning-based named entity recognition systems for bioentities, creating the GENIA corpus and initiating the ’Joint Workshop on Natural Language Processing in Biomedicine and its Applications’ (JNLPBA-2004) (Kim *et al.*, 2003). DNA, RNA, cell line and cell types were recognized in this project with a maximum F-score of 72.55% utilizing hidden Markov models (HMM) and support vector machines (SVM) (GuoDong and Jian, 2004a). Other participating systems utilized maximum entropy Markov models (MEMM) and conditional random fields (CRF).

The BioCreAtIvE challenges have also been playing a role in this development, with the first challenge focusing on gene recognition utilizing the GENETAG corpus, reporting a maximum F-score of 83.2% (Yeh *et al.*, 2005). Similar to the GENIA project, Markov models, SVMs, manually generated rules or a combination therefore were utilized. Since then, several publications have reported equivalent or improved performance, and several NER tools are currently available (Campos *et al.*, 2013a; Finkel *et al.*, 2005; McCallum, 2002; Settles, 2005).

The highest accuracies for open-source NER tools were reported by Gimli (Campos *et al.*, 2013a) at 72.23% overall F-score for JNLPBA corpus and 87.17% for the GENETAG corpus, using CRF-based models. This is comparable to the highest reported accuracies for these corpora with closed source software, where NERBio (Tsai *et al.*, 2006) report 72.9% for the JNLPBA corpus while (Hsu *et al.*, 2008) report 88.30% for the GENETAG corpus. These results for JNLPBA are also similar to others reported in literature (GuoDong and Jian, 2004b; Rei *et al.*, 2016). Genes and diseases were also reported to be identified with over 90% F-score by the NER module of DTMiner (Xu *et al.*, 2016). However, training and evaluation of the latter was performed on a custom corpus.

With the increase in popularity of neural networks, these have also been increasingly applied for biomedical NER (Crichton *et al.*, 2017; Gridach, 2017; Zeng *et al.*, 2017), improving on the state-of-the-art of traditional machine learning methods.

Despite these highly promising score values, there are a number of outstanding investigations and potential limitations which need to be considered and addressed: i) are the trained models generalizable and robust?; and therefore ii) is the high performance reported translatable?; iii) is performance limited by the size of the training data available?; and consequently, iv) would more annotated data improve the results?

Irrespective of the model utilized, machine learning NER approaches have often been trained and tested on a single corpus, frequently GENETAG or GENIA. This results in corpus-specific model optimizations, consequently introducing potential over-fitting which reduces model generalizability and reliability when applied to unseen text. This may be indicated from Campos *et al.* (2013b), where training for genes and proteins on the GENETAG corpus and testing on the CRAFT corpus achieved 45–55% F-score—lower performance when compared to the GENETAG test F-score of 87.17% by Gimli (Campos *et al.*, 2013a). The quality and different annotation standards of the different corpora may contribute to such a discrepancy in performance, however the variability in the style of writing of unseen text is also likely to increase when compared to the much smaller corpora, and thus the accuracy quoted for the models may not be representative. The difference between gold-standard performance and translational performance has indeed been previously shown for mutations (Caporaso *et al.*, 2008).

Several available corpora share the same/related entity classes: OSIRIS (Furlong *et al.*, 2008), SNPcorpus (Thomas *et al.*, 2011), BioInfer (Pyysalo *et al.*, 2007), various BioNLP 2011 subsets (Pyysalo *et al.*, 2012a), CellFinder (Neves *et al.*, 2012), GETM (Gerner *et al.*, 2010), IEPA (Ding *et al.*, 2001), HPRD50 (Fundel *et al.*, 2007), GREC (Thompson *et al.*, 2009) and GENIA (Kim *et al.*, 2003) all contain gene/protein-related entities; GENIA (Kim *et al.*, 2003), CellFinder (Neves *et al.*, 2012) and AnEM (Ohta *et al.*, 2012) contain cell line/type and tissue information; BioNLP2011 (Pyysalo *et al.*, 2012b), DDI corpus (Herrero-Zazo *et al.*, 2013) and GENIA (Kim *et al.*, 2003) share chemical/drug entities; and GENIA (Kim *et al.*, 2003), GREC (Thompson *et al.*, 2009), CellFinder (Neves *et al.*, 2012) and BioNLP2011 ID (Pyysalo *et al.*, 2012a) contain annotated species terms. Despite the common entities, availability of such data is very dispersed and formats and not standardized, varying from CONLL, to BioC, leXML and several others. Thus, here we collate a number of biomedically-related corpora currently available, convert relevant corpora to a common standard BioC format (Comeau *et al.*, 2013), and utilize multiple sources for training and testing of NER models to determine the effect of data size on evaluation and performance.

Performing this allows to generate corpus-independent models and determine if the current quantity of data available is enough to reach the maximum performance—a task commonly referred to as power analysis. Power analysis has been performed limitedly on NER systems, particularly biomedical NER, yet is a crucial part of evaluation to determine whether a system is bottlenecked by the data size, irrespective of algorithmic developments.

## 2 Materials and methods

### 2.1 Compiling and filtering corpora

Seventy-five biomedically-related corpora were compiled from primary or secondary sources. Annotation formats varied from standoff (.ann), IOB, BioC (Comeau *et al.*, 2013) or otherwise. Where multiple formats were available for the same corpus, all were compiled for cross-reference. The list of corpora compiled, the formats available and additional information such as year of publication and number of documents in corpus are listed in Supplementary Table S1. A similar table with the download links from the original or secondary host(s) is also provided on: https://github.com/dterg/biomedical_corpora/wiki.

Corpora which provide annotations of biomedical entities were considered for further processing. Corpora labeling entity relationships such as drug–drug interactions or protein–protein interactions were also considered relevant as long as the entities were explicitly annotated individually. Corpora with no annotation term indices provided, or with multiple nested entities were excluded, along with corpora annotating abbreviations. Subset corpora were also excluded when the superset corpus was available. For example: MLEE (Pyysalo *et al.*, 2012a) and AnEM (Ohta *et al.*, 2012) are subsets of the bigger AnatEM corpus (Pyysalo and Ananiadou, 2014), thus were excluded.

Details on subsequent format ’standardization’, processing and correction for annotation indexing mismatches are provided in Supplementary Methods.

### 2.2 Defining and remapping entity classes/ontologies

Different corpora annotate entities into different classes/labels. In order to merge different corpora with related entity classes, we devised and assessed eight initial super-classes based on ontologies: (i) ChemicalDrug; (ii) GeneProteinVariant; (iii) Cell; (iv) Anatomy; (v) Organism; (vi) Tissue; (vii) RNA; and (viii) Disease. Anatomy, tissue, cells, organisms and diseases are well-described, and their nomenclature is relatively static and consistent. In the case of organisms/species, their discovery requires mandatory registration, while for diseases these are documented in registers. Therefore, these entities are predicted to be well-recognized using dictionary matching approaches. Due to this, as well as the limited availability of unique training data other than the AnaTEM corpus, these classes will not be considered further here for machine learning training.

On the other hand, chemicals and drugs (particularly when mentioned using the IUPAC nomenclature), genes, proteins, RNA and especially mutations, are highly variable entities, with a greater possibility of mentioning non-previously-documented variants. These have thus been considered here for machine learning recognition. Based on preliminary results, some classes were further stratified. The original entity classes, the remapped classes and the number of entities for each corpus entity class are provided in Supplementary Table S2.

Different copies of the remapped corpora were devised, with one entity class per corpus copy. This was performed in order to allow for training and prediction of one entity class at a time. A single entity class classification was chosen over a multi-class classification for several reasons:

1. Scalability: with the availability of new corpora annotating new entity classes, recognizing the new entity class would require retraining the whole model in the case of an existing multi-class model. However with multiple single class models for each class, recognizing a new entity class would only require to train a new model for the new class and integrating with the existing models.
2. Multiple acyclic inheritance: An entity is not exclusive to one class and may thus belong to multiple classes. Classification and prediction in a multi-class model would not be straightforward with the current implementation. For example, on a single level, proteins are (a subset of) chemicals but not all chemicals are proteins, thus a protein entity belongs to both the class ‘proteins’ as well as ‘chemicals’ if these are considered separate.
3. Corpora available: training corpora available are highly varied; from specific corpora such as DDI (drug–drug interaction) corpus to broader chemical classes such as CHEMDNER which annotate chemicals including drugs and proteins into a single class.
4. Frontend: with the ultimate aim of providing a realistic evaluation and training of machine learning-based NER models for deployment in a scalable end-to-end tool, applications were considered. With single class classification models, an entity may have multiple annotations. This is favorable over a single annotation as if an entity such as ‘interleukin’ is listed and classified as only a chemical, a user will not recall it if querying proteins. Having it labeled as both a chemical and protein will allow for such entity to be recalled in both instances.

### 2.3 Model training and prediction

Several existing and stable NER packages utilize CRF-based models. Tools such as GIMLI (Campos *et al.*, 2013a), MALLET (McCallum, 2002) and Stanford NER (Finkel *et al.*, 2005) have been used widely and are commonly employed in end-to-end information extraction workflows such as the recent DTMiner (Xu *et al.*, 2016). Here we train and predict using the Stanford NER CRF algorithm based in Java (Finkel *et al.*, 2005).

To allow for an as fair as possible of a comparison with other tools, feature extraction methods were based on previous reports assessing the effect of features on performance by backward elimination. GIMLI (Campos *et al.*, 2013a) report that features such as: capitalization and symbols have a positive effect on performance (for the majority of entity classes), while Stanford report the increase in performance by disjunction and word tag features. The use and importance of character-level features, especially in the biomedical domain, has also been reported in neural network architectures (Gridach, 2017). These features were thus included as part of the feature extraction step. Additional sequence-related features were tested however these were determined to have no overall performance improvements. Details and commands used to perform model training and prediction are provided in the ‘model training and prediction’ section of the Supplementary Methods.

### 2.4 Power analyses

To determine the effect of training size on prediction performance, we performed power analyses to generate learning curves. Each corpus was split into 80% training and 20% test sets. Where applicable and possible, to avoid model bias, documents from the same manuscript were considered as either training or test. Prediction performance was measured by the F-score (Equation 1). The F-score was computed for each corpus rather than calculating an overall average. This provides an indication of which corpus is predicted the best and the worst and indicates any variation. To provide a single overall metric, two statistics were computed: (i) a document-weighted average was also calculated, where the F-score from each corpus is weighted by the number of documents it contains to compute an overall average; and (ii) an equally-weighted mean where corpora contributed equally to the overall average.

(1) $$Fscore=2\cdot \frac{Precision\cdot Recall}{Precision+Recall}$$

The learning curve was represented by an inverse power law function, previously reported (Figueroa *et al.*, 2012). Briefly, the prediction F-score ($Y_{fs}$) is defined as a function of the product of training sample size and minimum achievable error (a), learning rate (b) and decay rate (c) (Equation 2). An initial decay rate of -0.1 and learning rate of 0.2 were used. Error was defined as the root mean squared error (RMSE).

(2) $$Y_{fs}(x)=f(X;a,b,c)=(1-a)-b\cdot x^{c}$$

Learning curves were generated using three approaches:

- Corpus-specific training: To determine the model generalizability, we trained a model for each corpus and used this to predict the test data of other corpora;
- Merged corpora training: To determine the added value of increasing training size by integrating training data from multiple corpora, we merged/stacked the training data of all corpora and incrementally added the training data while predicting the same fixed test set;
- Leave-corpus-out training: To determine the generalizability of the merged-corpora-trained model, we trained a model using all training data of all corpora except one corpus, and tested the model by predicting the left-out corpus test data.

The addition of corpora was done using two approaches. In the first approach, when performing leave-corpus out, all corpora except one corpus were used for model training while the left-out corpus was used as ‘fixed’ test data. The ‘fixed’ test data was a random subset of all documents from each left-out corpus. For unbiased learning curve analysis, the data from the same corpus was excluded for model building. As for the training set, documents were randomly re-shuffled between corpora and added incrementally to the training data to obtain the learning curve. The leave-one-out corpora were predicted one at a time. In the second approach, when all corpora were used in the training, corpora were added sequentially, one document at a time. This determined how much added predictive value each corpus provides that is not captured by previously introduced training examples.

### 2.5 Orthographic feature analysis

To assess the distinguishing orthographic features between entity classes, we performed univariate tests on local morphological features. Labeled entities for each class of interest were extracted and labeled accordingly, and the rest of the tokens were considered ’nulls’. These two classes were balanced by random sampling from the bigger class. GIMLI (Campos *et al.*, 2013a) and regular expressions were used to extract a total of 31 morphological features for these tokens, including: different forms of punctuation, case sensitivity (initial caps, end caps, all caps), digits (number of digits) and number of characters.

Each feature was represented by a binary representation for each token and therefore summation provided total occurrence of a feature in the ‘null’ class and ‘entity’ class. This allowed for the calculation of the percentage difference of a feature occurrence between the classes. Statistically significant feature differences were determined by performing Fisher exact tests followed by Benjamini-Hochberg FDR-correction for multiple testing. This was repeated n-times [where *n* = size(largest class)/size(smaller class)] and a mean *q*-value ± standard deviation was computed for each feature. When a feature was determined to be significantly different between the classes (*q* < 0.05; including the upper deviation boundary), and the percentage difference was positive between the entities class and the null, such feature was considered ‘characteristic’ of that class (compared to null tokens; Fig. 3).

## 3 Results and discussion

### 3.1 Identifying genes and proteins

Ontologically, proteins, genes and variants are related. These were initially merged in a single superclass to test the overlap between the annotated entities across different corpora. SNPcorpus and tmVar achieved almost no predictive performance prior to the introduction of OSIRIS/SETH training data (Supplementary Fig. S1). SNPcorpus and tmVar annotate mutations, while none of the training corpora prior to the introduction of OSIRIS/SETH data have mutations annotated. While expected, this confirms that mutation entities are significantly different from the gene/protein classes and were thus considered as a separate class in subsequent tasks. Contrastingly, while VariomeCorpus also annotates variants, this corpus also annotates genes, with 1690 mutant entities and 4613 genes. This explains why VariomeCorpus test data was better predicted in comparison to mutation-specific corpora SNPcorpus and tmVar. The difference between the GeneProtein class and variants is more evident when considering the orthographic features (Fig. 3), where genes and proteins from different corpora share several univariate features, but less so with entities in the variant class.

**Fig. 1.**
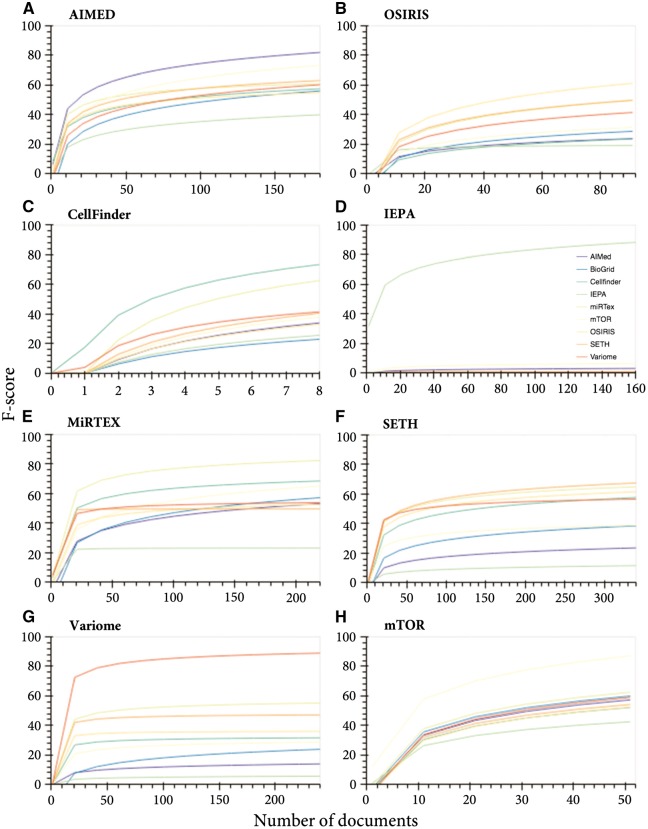
Corpus-specific learning curves for the ‘GeneProtein’ class. Learning curves for corpus-specific training and prediction of all corpora test data. (**A**) AIMED, (**B**) OSIRIS, (**C**) CellFinder, (**D**) IEPA, (**E**) MIRTEX, (**F**) SETH, (**G**) VariomeCorpus and (**H**) mTOR

To determine the generalizability of the ‘GeneProtein’ class models, when excluding variants, we tested the cross-performance of models trained on corpora individually and applied it to other corpora (not seen in the training—leave corpus out cross validation) (Fig. 1). While increasing training data increased performance in all cases, the best predictions of the test data were achieved when the test data originated from the same corpus, with varying predictive capacity for other corpora (Fig. 1A–H). Furthermore, IEPA data was the hardest to predict (Fig. 1A–C; E–H), and inversely, IEPA-trained model was unable to predict any of the other test data for the other corpora (Fig. 1D), suggesting data incompatibility or corpus bias. Considering the orthographic features on their own, IEPA was indeed the most inconsistent compared to other GeneProtein corpora (Fig. 3), with the IEPA corpus having very different ‘fingerprint’ of significant features compared to other corpora within the same class.

Merging the different corpora for both training and testing increased the consistency and overall performance for the GeneProtein class (Fig. 1A). With respect to sample size dependence, increasing the training data generally incrementally improved the performance. Some corpora left out from training were predicted by other corpora with similar performance to their own; for example, SETH was predicted with an F-score of 64% when all corpora were merged for training, while leaving SETH out of training obtained a 63% F-score. In case of miRTex, both merged training data and all corpora other than miRTex, converged at an F-score of 76% at 450 documents, although additional documents improved the performance of the former up to 84% F-score.

Generally, the maximum F-score for the test data of a specific corpus was only achieved when introducing training data from the same corpus. Nonetheless, in most cases, a relative performance plateau is reached after 1000 training documents, with a maximum weighted average of 78.32% F-score (Supplementary Fig. S2).

### 3.2 Identifying variants

Based on the predictive power and orthographic differences, variants were considered as a class on their own, despite the similarity in ontology and semantics. When predicting the test data of a corpus by other corpora (leave-corpus-out cross-validation), VariomeCorpus was poorly predicted by any corpus. Taking a closer look at the raw corpus, it appears that most entities are genes followed by the token ‘mutant’ rather than mutation entities following the standard nomenclature. With the entity structure being ‘gene X mutant’, this possibly explains why entities in the VariomeCorpus test data were identified and predicted as genes in the ‘GeneProteinVariant’ superclass (Supplementary Fig. S1). Once again, this difference is highlighted by the orthographic feature map (Fig. 3) where VariomeCorpus has 4 significant features (OneDigit, OneCap, ThreeCap and Length3-5) which are not shared with any of the other ‘variant’ corpora. Inversely, whereas all other variant corpora were identified to have the ‘+’ character as significant (a symbol commonly used to denote mutations), VariomeCorpus was the only not to share such characteristic. Indeed, VariomeCorpus was recently reported to annotate many vague mentions such as ‘de novo mutation’ and ‘large deletion’, with only a subset mentioning position-specific variants (Cejuela *et al.*, 2017). Due to such differences, this was excluded from subsequent power analyses. However, if more training data is required, this subset can be identified and extracted, as described earlier (Jimeno Yepes and Verspoor, 2014).

When merging data from SETH, SNPcorpus, tmVar and OSIRIS, SETH and tmVar test data was predicted with $\tilde{8}$2% in both cases, plateauing around 500 documents. tmVar test data was predicted with equal performance when performing leave-corpus-out cross-validation. Contrastingly, the predictive capacity of other corpora on unseen corpora test data as well as when using merged training data was very low (Fig. 1B). This suggests a subset of the entities are ’unique’ in these corpora. OSIRIS obtained the lowest plateaued performance, and by looking at OSIRIS annotations, indeed they appear to contain non-standard nomenclature, with annotations such as: ‘codon 72 (CCC/proline to CGC/arginine’, ‘(TCT TCC) in codon 10’, ‘-22 and -348 relative to the BAT1 transcription start site’, ‘A at positive -838’, ‘C in -838’. However, these are still valid mutation-related entities, thus to recall such entities, more training data similar to OSIRIS is required, although standardization of nomenclature in more recent publications may render this unnecessary. Nonetheless, when omitting OSIRIS and re-plotting SETH, SNPcorpus and tmVar learning curve, SETH and tmVar obtained lower performance, backing up the positive contribution of OSIRIS training data to the predictive performance of tmVar and SETH.

**Fig. 2.**
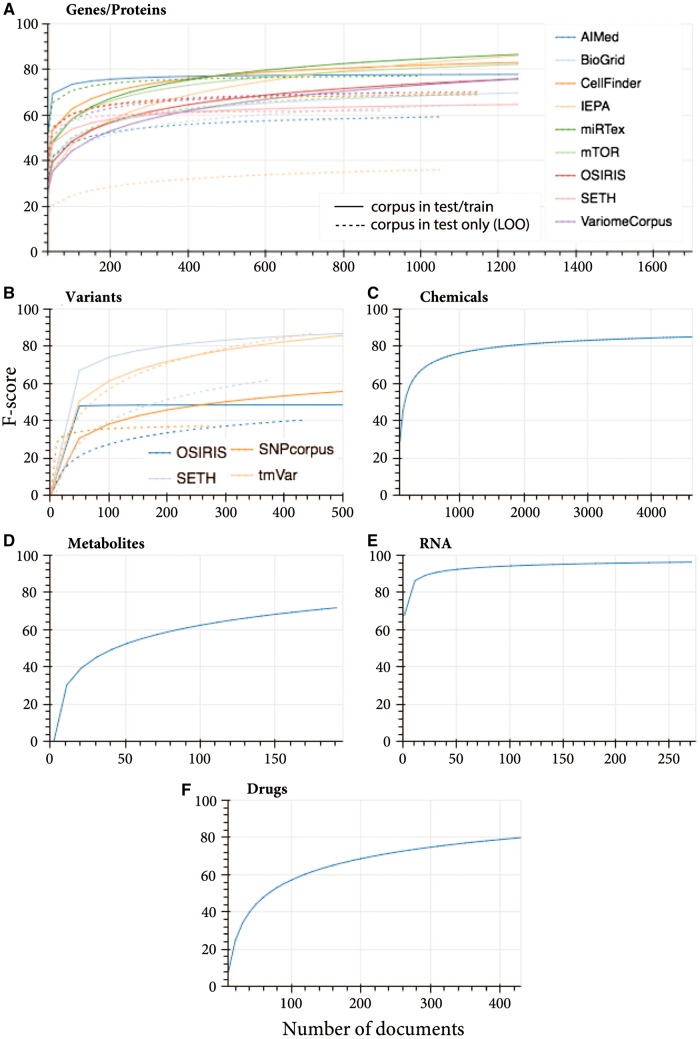
Learning curves for merged training data from multiple sources and prediction of the test data for each corpus individually, and leave-corpus-out cross-validation where each corpus is left out from training and its test data is predicted by all other corpora (where multi-source data is available). Training and testing of the classes: (**A**) genes and proteins (dashed lines represents leave-one-out prediction learning curves), (**B**) variants, (**C**) chemicals (CHEMDNER corpus), (**D**) metabolites (Metabolites corpus), (**E**) RNA (miRTex corpus) and (**F**) drugs (DDIcorpus)

Looking at the corpus-specific learning curves (Supplementary Fig. S3), tmVar training data only (Supplementary Fig. S3C) predicts tmVar test data with ∼50%. The trend of the learning curve indicates that with more training data the performance is expected to increase. Indeed, the performance increased to >80% when training data from other corpora was added (Fig. 2B). Leaving the tmVar training data out completely and using the other corpora to predict tmVar test data achieved the same performance, indicating high model generalizability (Fig. 2B).

SETH achieved the same performance (86.02% F-score) when merged training data (Fig. 2B) and SETH only training data were used (Supplementary Fig. S3B). Leaving out SETH training data achieves lower F-score (67.09%), therefore the SETH corpus alone is contributing to 18.93% additional performance. With respect to generalizability, SETH predicted 66.67% of tmVar test data (Supplementary Fig. S3B), and considering the maximum tmVar performance was achieved even when tmVar train data was omitted, the remaining performance was achieved by the training data of other corpora (SNPcorpus and OSIRIS).

SNPcorpus and OSIRIS achieved a similar trend in performance when merging corpora (Fig. 2B) and when using corpus-specific training data (Supplementary Fig. S3D and A). The absolute performance is slightly lower in the former case, suggesting introduction of noise to the model.

**Fig. 3.**
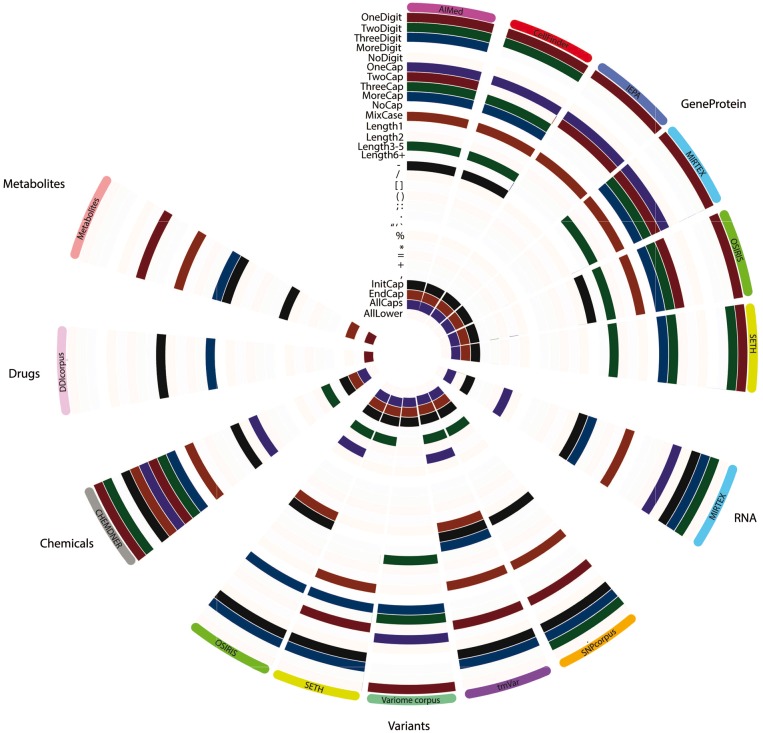
Orthographic feature analysis for entity classes determined per corpus. Features highlighted were identified to be univariately significant for an entity class in a given corpus. Each layer/row represents an orthographic feature while each column represents a corpus, grouped by entity classes to represent six main classes: GeneProtein, RNA, variants, chemicals, drugs and metabolites

With respect to orthographic features, the ‘variants’ class is quite variant across different corpora (Fig. 3B), with very limited consistently significant features across corpora. Commonly, three or more digits are present in ‘variant’ entities, however overall there is no distinctly evident ‘fingerprint’ of univariate significant features across the different corpora. This variation has been explored in detail by Cejuela *et al.* (2017), where mutation mentions have been classified as ‘standard’, ‘semi-standard’ and ‘natural language’, with SETH and tmVar sharing a subset of standard mutations while only SETH captured natural language mentions (Cejuela *et al.*, 2017).

### 3.3 Identifying chemicals, drugs and metabolites

Based on ontology, corpora annotating chemicals, drugs and metabolites were remapped into a single ‘ChemicalDrug’ superclass. However, corpora such as CHEMDNER annotate genes as chemicals while more specific corpora such as DDI and metabolites corpus only annotate a particular entity class: drugs and metabolites respectively, thus would not be able to predict genes in the test set. Given these annotation mismatches between corpora, we devised new classes. CHEMDNER is the biggest corpus with over 58 000 chemical entities annotating formulae and multiple alternative names such as: systematic names and chemical families. This comprehensive, large and highly diverse naming system is not found in any other corpora and hence this corpus was considered on its own. Since genes/proteins such as ‘interleukin-2’ are also considered as an entity in this corpus, such entity would be annotated multiple times when the GeneProtein model and CHEMDNER model are used to predict its class. This is reasonable considering that such entity indeed can be considered as a child of the GeneProtein parent as well as the Chemical superclass. This backs up the reason why single entity class models (binary class classification problems) were considered in this study (compared to multi-class models). With the new classes, chemicals, metabolites and drugs could only be trained on single corpora. A stable performance was achieved after 1200 training documents for chemicals, 160 documents for metabolites and 400 documents for drugs.

CHEMDNER achieved 84.8% F-score when all training documents are utilized during model training (Fig. 2C), with performance stabilizing around 1500 documents. This is similar to the performance published by the authors (Krallinger *et al.*, 2015).

When devising a model for drug NER, while DDIcorpus and mTor both contain drug annotations, mTor only annotates three unique drug entities (Supplementary Table S2) and hence was excluded. The drug learning curve for DDIcorpus indicates a stable performance at and beyond 380 documents, with an average of 78.48% (Fig. 2F).

Similarly, metabolites corpus is the only resource that specifically annotates metabolites. Other corpora such as CHEMDNER annotate metabolite entities, however these are labeled under a broad chemical class hence cannot be distinguished from other non-metabolite entities such as drugs and proteins. The learning curve for the metabolites corpus was generated and is shown in Figure 2D. Prediction performance stabilizes around 160 documents with an average F-score of 71.98%.

### 3.4 Identifying RNA

As listed in Supplementary Table S2, RNA is annotated in miRTex and mTor corpora. While miRTex contains over 2700 entities, mTor only annotates 7 unique entities and thus the latter was excluded as this is insufficient for representative power calculations. Furthermore, with such small number of entities, any performance metrics would not provide meaningful insight following train/test split, especially with regards to model generalizability.

MiRTex corpus achieved a plateaued performance of 91% with 21 documents (Fig. 2), which increases marginally to a stable F-score of 96.17%. This high performance and stability may be accounted for by the high consistency in RNA nomenclature.

## 4 Conclusion

A generalizable model is crucial for applied machine learning-based named entity recognition. Here we show that merging training data from multiple sources may generate a more generalizable model. However, the absolute performance may be lower when comparing it to individual source-trained models due to annotation standard differences, as well as corpus over-fitting of the latter. Overfitting to one corpus may be the case when the corpus is a selection of publications from a medical subfield (e.g. cardiac diseases) and therefore the corpus is not representative of the class. The average performance achieved for genes and proteins for a merged data model is comparable to existing models, while variants showed high variability. Training data for chemicals, drugs, metabolites and RNA is limited due to lack of overlap of entity types across corpora, however individual models show no increased performance with increasing training data. Generally, collecting more training data is unlikely to increase performance of bioentity named entity recognition. However, with improved annotation standards and implementation of transfer learning approaches may not only improve performance but also generalizability—providing a more realistic performance measure of translational named entity recognition.

## Funding

The authors acknowledge the financial support for bioinformatics developments as part of BBSRC (BB/L020858/1) and EU-METASPACE (34402) projects; DG acknowledges Imperial College Stratified Medicine Graduate Training Programme in Systems Medicine and Spectroscopic Profiling (STRATiGRAD); KV and DG acknowledge Waters corporation for funding and support throughout this study.

*Conflict of Interest*: none declared.

## Supplementary Material

Supplementary Data
