# Supplementary material for: Exploiting and assessing multi-source data for supervised biomedical named entity recognition
Source: Bioinformatics. 2018 Mar 10;34(14):2474–82. doi: 10.1093/bioinformatics/bty152 (PMC6041968; doi:10.1093/bioinformatics/bty152)
Supplement: Supplementary Data [file bty152_supp-info-exploiting.pdf]

Supplementary Information

Supplementary Methods

Format Conversion and Annotation Correction

CRF-based models are the most widely adopted and documented approaches in end-to-end biomedical NER tools, thus for comparative purposes, we adopted the approach. Stanfords NER module (Finkel et al., 2005) was used to train a CRF model, requiring training data in IOB2 format, where the initial token in multi-term entity is labeled as B-LABEL (B for "Beginning"), and internal tokens following the B-LABEL are labeled as I-LABEL (I for "Inside") (Krishnan and Ganapathy, 2005). All other null tokens are labeled as "O". However, while required for training, such format is tokenization-dependent and loses article information, if this is required for further validation and transparency. To retain all annotation information from the original corpus, such as: document source, passage and annotation position, selected corpora in the BRAT format were initially converted to the BioC format standard (Comeau et al., 2013) using the available Brat2BioC Java module (Yepes et al., 2013). The provided annotation indices were in turn checked for errors: an offset/mismatch between 1-5 characters was corrected automatically, while larger offsets were manually validated and corrected.

Corrected and BioC-converted corpora were finally converted to IOB2 using a custom python script and the python pyBioC library (Marques and Rinaldi, 2013). Unless a custom DTD (Document Type Definition) was used and provided by the corpus (as for tmVar corpus (Wei et al., 2013)), the default DTD was used to process BioC documents. As part of the conversion, following sentence tokenization, word tokenization was carried out using the NLTK regular expression tokenizer with the expression: "`\w+|[\S\w]`". This was chosen over other python NLTK tokenization methods as 'TreebankTokenizer', 'WordPunctTokenizer', 'PunktWordTokenizer', and 'WhitespaceTokenizer' as these contract some (or all) of the punctuation, creating a token with embedded punctuation which does not match the entity/annotation when the annotation is part of a punctuated token. For example: "[...] gene X-associated [...]" tokenizes to "gene" and "X-associated" using "TreebankWordTokenizer" and "PunktWordTokenizer", however the gene entity in this case is only "X" or "gene X". In the case of a terminal entity (e.g. "[...] gene X."), the punctuation is contracted using the "PunktWordTokenizer".

Model Training and Prediction

A python wrapper was developed to train and predict data using the Stanford Core NLP Java toolbox, by executing the following shell commands:

Training: javacp stanford-ner.jar;lib/*;. edu.stanford.nlp.ie.crf.CRFclassifier prop trainPropFile

Prediction: javacp stanford-ner.jar;lib/*;. edu.stanford.nlp.ie.crf.CRFclassifier -loadClassifier trainedModel prop testPropFile

Training and test data are provided through a file list in the properties file (.prop file). The train and test prop files are modified and populated prior to running the model by inserting the list of train and test files. Alternatively, train and test files can be inputted as an argument to the command line, however given that this is limited by the number of

characters in the input shell command (imposed by the operating system used), and the number of files used for training exceeded such limit, we opted for the modification of the .prop file.

Table S1: List of compiled biomedical corpora, their original format, year of publication, and size of data. Number of documents for each corpus may vary based on the source, and a document unit may be defined differently in different corpora (e.g. abstract, title, whole manuscript text). The sources from which these each of these are available and were originally obtained are provided on: `https://github.com/dterg/biomedical_corpora/wiki` and `https://bitbucket.org/iAnalytica/bioner`, where sources may be the original manuscript published, or if not available (or available in a different formation), other secondary sources hosting the resource. When a corpus is available in various formats and multiple sources, these are indicated.

Corpus	Year	Format	Documents
Ab3P (Abbreviation Plus P-Precision)	2008	BioC	1250 PubMed Abstracts
AIMed	2005	BioC	$\sim$ 1000 MEDLINE abstracts (200 abstracts)
AnatEM (Anatomical entity mention recognition)	2013	CONLL, standoff	1212 docs (500 docs from AnEM + 262 from MLEE + 450 others)
AnEM	2012	BioC	500 docs (PubMed and PMC); abstracts and full text drawn randomly
AZDC (Arizona Disease Corpus)	2009	IeXML, .txt	2856 PubMed abstracts (2775 sentences). Other source says 794 PubMed Abstracts
BEL (BioCreative V5 BEL Track)	2016	BioC	
BioADI	2009	BioC	1201 PubMed abstracts
BioCause	2013	standoff	19 full-text documents
BioCreative-PPI		XML	
BioGRID	2017	BioC	120 full text articles
BioInfer	2007	BioC	1100 sentences from biomedical literature
BioMedLat	2016	standoff	643 BioASQ questions/factoids
BioText	2004	txt	100 titles and 40 abstracts
CDR (BioCreative V)		BioC	
CellFinder 1.0	2012	BioC	10 full documents from PMC from (Loser et al. 2009) on ”Human Embryonic Stem Cell Lines and Their Use in International Research”
CG Cancer-Genetics (BioNLP-ST 2013)	2013	BioC, standoff	
CHEMDNER (BioCreative IV Track 2)	2013	BioC / standoff	

Chemical Patent Corpus	2014	standoff	200 patents
CoMAGC	2013	XML	821 sentences on prostate, breast and ovarian cancer
CRAFT	2012		97 full OA biomedical articles
Craven (Wisconsin corpus)	1999	other	1,529,731 sentences (automated)
CTD (BioCreative IV Track 3)		BioC	
DDICorpus	2011 2013	BioC	792 texts from DrugBank and 233 Medline abstracts
DIP-PPI (Database of Interaction Proteins)		other	Only proteins from yeast.
EBI:diseases	2008	other	856 sentences from 624 abstracts
eFIP	2012 2015	xlsx	
EMU (Extractor of Mutations)	2011	other	
EU-ADR	2012	other	300 PubMed abstracts (drug-disoder, drug-target, gene-disorder, SNP-disorder)
Exhaustive PTM (BioNLP 2011)			
FlySlip	2007	CONLL	82 abstracts, 5 full papers
FSU-PRGE	2010	leXML	3236 MEDLINE abstracts (35,519 sentences)
GAD	2015	csv	
GeneReg	2010	BioC	314 Abstracts
GeneTag (BioCreative II Gene Mention)	2005	BioC	20,000 sentences MEDLINE
GENIA (BioNLP Shared Task 2009)			
GENIA (BioNLP Shared Task 2011)		BioC, standoff	
GENIA (term annotation)	2003	BioC, XML	
GETM	2010	BioC, standoff	
GREC (Gene Regulation Event Corpus)	2009	BioC, standoff, XML	240 MEDLINE (167 on E.coli and 73 on Human)
HIMERA	2016	standoff	
HPRD50 (Human Protein Reference Database)	2004	BioC	50 abstracts
IDP4+	2017	anndoc	826 abstracts/full texts
IEPA	2002	BioC	slightly over 300 MEDLINE abstracts
iHOP	2004	other	$\sim$ 160 sentences

iProLINK / RLIMS	2004	other, XML, BioC	
iSimp	2014	BioC	130 MEDLINE abstracts (1199 sentences)
Linnaeus	2010	standoff	
LLL (Learning Language in Logic)	2005	BioC	
MEDSTRACT		BioC	199 PubMed citations
MedTag	2005	other	
Metabolite and Enzyme	2011	BioC, XML	296 abstracts
miRTex	2015	BioC, standoff	350 abstracts (200 development, 150 test)
MLEE	2012	CONLL, standoff	262 PubMed abstracts on molecular mechanisms of cancer (specifically relating to angiogenesis)
mTOR pathway event corpus (BioNLP 2011)	2011	standoff	
MutationFinder	2007	other	305 abstract (development data set), 508 abstract test set
Nagel		XML, standoff	
NCBI Disease	2012	other	6881 sentences in 793 PubMed abstracts
OMM (Open Mutation Miner)	2012	other	40 full texts
OSIRIS	2008	BioC, XML, standoff	105 articles
PC (Pathway Curation) (BioNLP-ST 2013)	2013	BioC	
PennBioIE-oncology	2004	leXML	1414 PubMed abstracts on cancer
pGenN (Plant-GN)	2015	BioC	104 MEDLINE abstracts
PICAD	2011	XML	1037 sentences from PubMed
PolySearch (includes v1. and v2.)		other	
ProteinResidue		other	
SCAI_Klinger	2008	CONLL	
SCAI_Kolarik	2008	CONLL	
SETH	2016	standoff	630 publications from The American Journal of Human Genetics and Human Mutation
SH (Schwartz and Hearst)	2003	BioC	1000 PubMed Abstracts
SNPCorpus	2011	BioC	296 MEDLINE abstracts
Species	2013	standoff	800 PubMed abstracts
T4SS (Type 4 Secretion System)	2011	CONLL	

T4SS Event Extraction (BioNLP 2010)	2010	other	
tmVar	2013	BioC	500 PubMed abstracts
VariomeCorpus (hvp)	2013	BioC	
Yapex	2002	other	99 training, 101 test MEDLINE abstracts

Table S2: Statistics for the original corpora considered for model training and testing, their respective original entity classes, total number of entities, number of unique entities, and their remapping into new entity classes.

Corpus	Entity Class	Entity class (remapped by ontology)	Number of entities	Number of unique entities
AIMED	protein	GeneProtein	4236	1138
BioGrid	Gene	GeneProtein	6489	1068
CellFinder	GeneProtein	GeneProtein	1750	734
VariomeCorpus	gene	GeneProtein	4613	453
IEPA	Protein	GeneProtein	1117	130
	Gene	GeneProtein	1266	484
miRTex development	Complex	GeneProtein	24	7
	Family	GeneProtein	57	28
	Gene	GeneProtein	922	368
miRTex test	Complex	GeneProtein	32	9
	Family	GeneProtein	78	31
	Tag	GeneProtein	3	2
mTor	Receptor	GeneProtein	1	1
	Protein	GeneProtein	1483	297
	Complex	GeneProtein	201	69
OSIRIS	gene	GeneProtein	799	260
SETH	Gene	GeneProtein	2315	969
VariomeCorpus	mutation	Variants	1690	429
OSIRIS	variant	Variants	551	369
SETH	SNP	Variants	895	689
	RS	Variants	9	3
SNPCorpus	NSM	Variants	244	230
	PSM	Variants	278	216
	SNP	Variants	39	29
tmVar test				

	ProteinMutation	Variants	205	137
	DNAMutation	Variants	220	156
	SNP	Variants	96	58
tmVar train	ProteinMutation	Variants	440	254
	DNAMutation	Variants	431	305
	MULTIPLE	ChemicalDrug	188	175
	NO CLASS	ChemicalDrug	32	15
	FAMILY	ChemicalDrug	4223	1573
	ABBREVIATION	ChemicalDrug	4521	812
CHEMDNER - Development	SYSTEMATIC	ChemicalDrug	6813	2756
	FORMULA	ChemicalDrug	4137	839
	IDENTIFIER	ChemicalDrug	639	240
	TRIVIAL	ChemicalDrug	8970	2268
	MULTIPLE	ChemicalDrug	202	177
	NO CLASS	ChemicalDrug	40	13
	FAMILY	ChemicalDrug	4086	1444
	ABBREVIATION	ChemicalDrug	4536	822
CHEMDNER - Training	SYSTEMATIC	ChemicalDrug	6655	2820
	FORMULA	ChemicalDrug	4448	840
	IDENTIFIER	ChemicalDrug	672	231
	TRIVIAL	ChemicalDrug	8823	2172
DDI - negative	DrugName	ChemicalDrug	826	416
DDI - positive	DrugName	ChemicalDrug	240	176
VariomeCorpus	Chemicals_Drugs	ChemicalDrug	1	1
Metabolites	Entity	ChemicalDrug	2454	653
	Ion	ChemicalDrug	5	2
mTOR	Simple_molecule	ChemicalDrug	26	13
	Drug	ChemicalDrug	42	3
miRTex - development	MiRNA	RNA	1539	469
miRTex - test	MiRNA	RNA	1217	353

mTOR	RNA	RNA	12	7

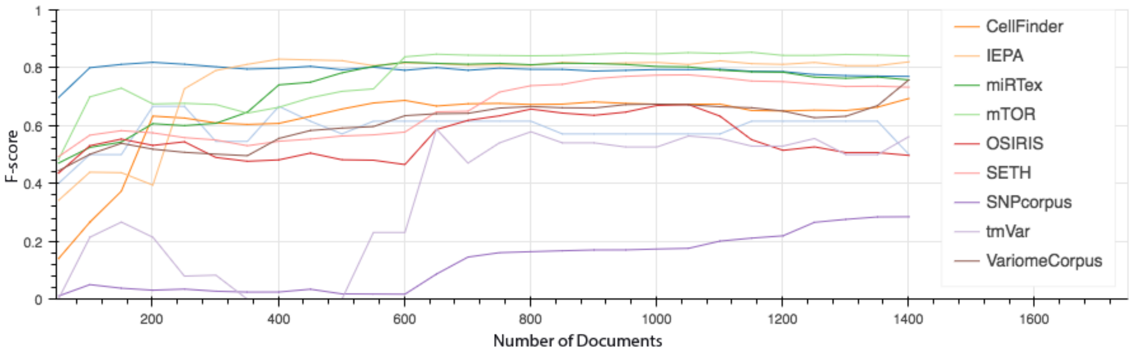

Figure S1: Raw learning curves obtained when considering genes, proteins and variants as a single superclass. Although different corpora may have differences in annotation standards for the same entities, it can be noted that the overall predictive performance of the trained models does not decrease.

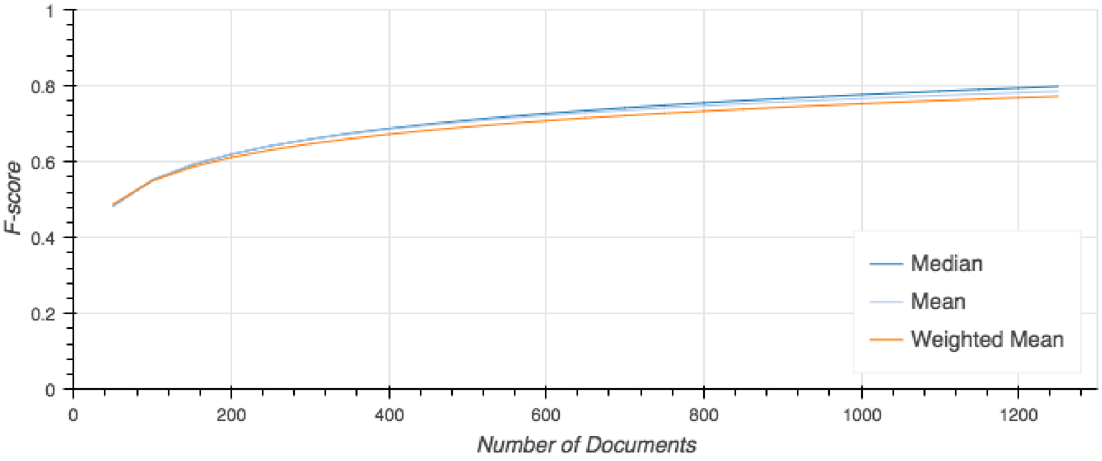

Figure S2: Average performance for the "GeneProtein" prediction when using all corpora for training. All relevant corpora were merged and split for training and testing. The average (mean, median and weighted mean aggregated F-score prediction performance for "Gene-Protein" superclass entities is shown, increasing incrementally with increasing document training size.

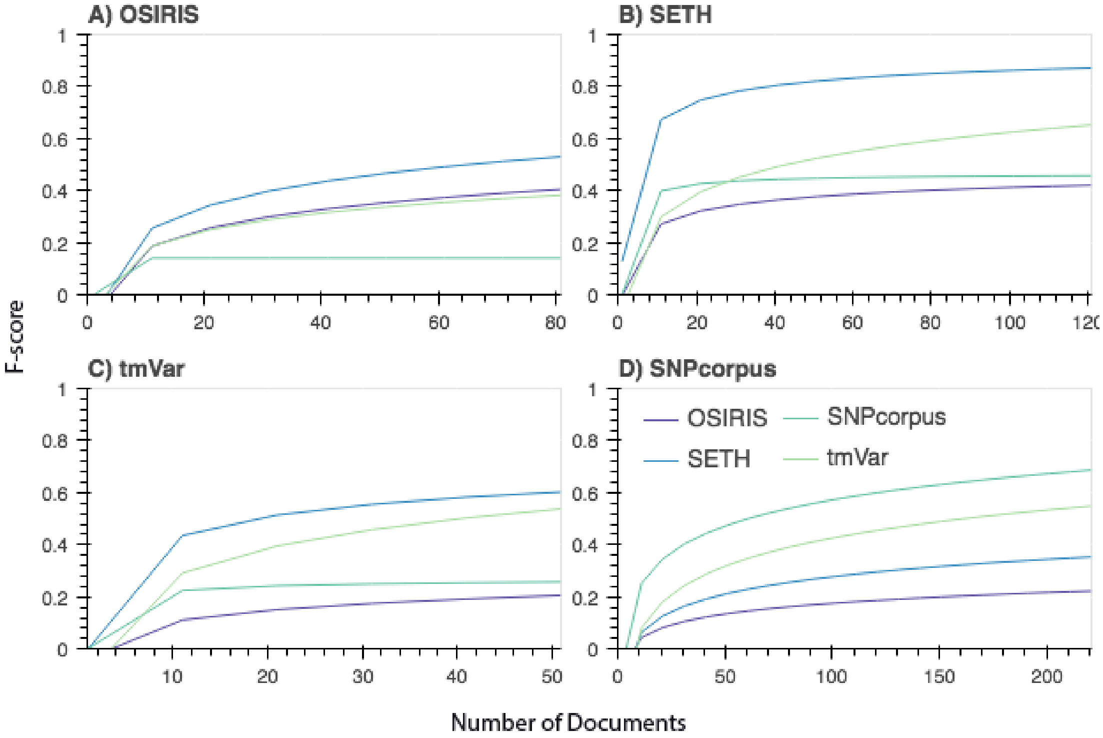

Figure S3: Corpus-specific learning curves for the “Variants” class. Learning curves for corpus-specific training and prediction of all corpora test data. A) OSIRIS; B) SETH; C) tmVar; and D) SNPcorpus

References

Donald C. Comeau, Rezarta Islamaj Doan, Paolo Ciccarese, Kevin Bretonnel Cohen, Martin Krallinger, Florian Leitner, Zhiyong Lu, Yifan Peng, Fabio Rinaldi, Manabu Torii, Alfonso Valencia, Karin Verspoor, Thomas C. Wiegers, Cathy H. Wu, and W. John Wilbur. Bioc: a minimalist approach to interoperability for biomedical text processing. *Database*, 2013:bat064, 2013. doi: 10.1093/database/bat064. URL http://dx.doi.org/10.1093/database/bat064.

Jenny Rose Finkel, Trond Grenager, and Christopher Manning. Incorporating non-local information into information extraction systems by gibbs sampling. In *Proceedings of the 43rd Annual Meeting on Association for Computational Linguistics*, ACL '05, pages 363–370, Stroudsburg, PA, USA, 2005. Association for Computational Linguistics. doi: 10.3115/1219840.1219885. URL https://doi.org/10.3115/1219840.1219885.

Vijay Krishnan and Vignesh Ganapathy. Named entity recognition, 2005.

Hernani Marques and Fabio Rinaldi. Pybioc: a python implementation of the bioc core. In *Fourth BioCreative Challenge Evaluation Workshop*, volume 1, pages 2–4. Biocreative, October 2013. URL http://www.zora.uzh.ch/id/eprint/91881/.

Antonio Jimeno Yepes, Mariana Neves, Karin Verspoor, and formats. Brat2bioc: conversion tool between brat and bioc. 2013.
